# Clinical translation section: accelerating the pace from bench to bedside

**DOI:** 10.1186/1479-5876-9-116

**Published:** 2011-07-21

**Authors:** Lana E Kandalaft, George Coukos

**Affiliations:** 1 Ovarian Cancer Research Center, University of Pennsylvania, Philadelphia, PA 19104, USA

## Editorial

Cell and gene therapy clinical trials have been conducted for various indications such as cancer, cardiovascular diseases and autoimmune disorders for more than 20 years. The increased understanding of immune function, cancer biology, and stem cell biology have dramatically accelerated the development of technology for cell and gene therapy in these areas. Supported by some successful clinical results, the development of many potential new technologies has produced an explosion of therapeutic pursuits in the clinic. The new technologies have produced significant challenges in the clinical translation space, including the need to develop innovative clinical trial designs, to accelerate development of therapies by minimizing the number of patients required to evaluate safety and efficacy; to develop and incorporate methods to capture important biologic effects of cell and/or gene based therapies in patients; and to dissect the impact of therapeutic combinations. Furthermore, the increasingly personalized flavor of cell and gene therapies has produced an ever-greater need for developing reliable biomarkers for selecting patients and measuring biologic effects of therapy. Finally, regulatory agencies have recognized the need for modifying acceptable evaluation metrics to respond to the increasing complexity in clinical design and interventions. Yet, despite significant advancement in the field, progress remains slow relative to discovery and the need of speeding up clinical application of basic science discoveries is still unmet. Indeed, the rapid advancement of therapeutic technologies in the laboratory; the plethora of biomarker candidates; and the recent innovations in clinical science concerning trial design, contrast with the slow development process from conception of an idea till proof of concept in the clinic. Typically, clinical trials of this nature take several years to develop, implement, complete and report. This creates a significant gap of knowledge in the field, as clinical innovation takes several years to be communicated.

The incessantly evolving field of cell and gene therapy requires early rapid and extensive communication to ensure continuing progress. The goal of JTM 'Clinical Translation Section' is to provide a new space for the rapid communication of innovative early phase clinical trials, where novel scientific ideas are translated to the clinic, at the time of initiation of a clinical study. These include investigator-initiated as well as industry-sponsored clinical trials, which have completed the regulatory process and are about to start accruing patients. No clinical results are required. These "white papers" review the background, rationale, clinical design and approach, translational endpoints and expected outcomes of a new clinical trial in a scholarly manner, and provide the opportunity to investigators to share their views and clinical translation efforts with a wide audience at an early stage, which could benefit greatly other investigators in the field. Such therapies will target a range of diseases including cancer, cardiovascular diseases, autoimmune disorders or other common or rare diseases where cell-based, gene-based, biological or otherwise targeted therapy is applied. A broad-based and multidisciplinary editorial board stemming from academia, biotech and pharma with expertise in cancer immunotherapy; cellular manufacturing; gene therapy; stem cells; regenerative medicine; cardiovascular medicine; and autoimmunity will evaluate manuscripts. Accepted manuscripts will be distinguished for the novelty in approach, design or clinical indication of the study and unique ability to translate laboratory concepts from the bench to the bedside. Lastly, this section will also provide a platform for earlier academic recognition of the significant efforts of translational clinical investigators whose careers primarily depend on the execution of clinical trials.

